# Diagnosis of vocal cord dysfunction / inducible laryngeal obstruction—A Delphi study protocol

**DOI:** 10.1371/journal.pone.0279338

**Published:** 2022-12-29

**Authors:** Paul Leong, Anne E. Vertigan, Mark Hew, Malcolm Baxter, Debra Phyland, James H. Hull, Thomas L. Carroll, Peter G. Gibson, Vanessa M. McDonald, Philip G. Bardin

**Affiliations:** 1 Monash Health, Melbourne, Victoria, Australia; 2 Monash University, Melbourne, Victoria, Australia; 3 John Hunter Hospital, Newcastle, Australia; 4 Centre of Excellence in Treatable Traits, University of Newcastle, Newcastle, Australia; 5 Alfred Hospital, Melbourne, Victoria, Australia; 6 Royal Brompton Hospital, London, United Kingdom; 7 Brigham and Women’s Hospital, Boston, Massachusetts, United States of America; Universidade de Sorocaba, BRAZIL

## Abstract

**Introduction:**

Currently there is no consistent and widely accepted approach to the diagnosis of vocal cord dysfunction/inducible laryngeal obstruction (VCD/ILO). Harmonised diagnostic methods are vital to enable optimal diagnosis, advance management and enable research. We aim to obtain consensus on how expert clinicians recognise and diagnose VCD/ILO.

**Methods and analysis:**

Two-round modified Delphi, with workshop validation.

**Ethics and dissemination:**

Institutional Board Review was obtained from the Monash Health Human Research Ethics Committee. The dissemination plan is for presentation and publication.

**Registration details:**

Registered at Australia and New Zealand Clinical Trials Registry ACTRN12621001520820p.

## Introduction

In 1842, Robley Dunglison described a “spasmodic affection of laryngeal muscles” resulting in “crowing inspiration” and dyspnoea [1]. Subsequent reports emphasized specific aspects of the presentation including upper airway obstruction [2], an association with airways disease [3–5] and psychological disorders [6]. The condition is characterised by unexplained breathlessness accompanied by vocal cord closure and may occur in healthy individuals as well as being associated with asthma and other lung diseases [7]. Importantly, clinical experience has emphasised that VCD/ILO is a heterogenous condition and likely to contain several ‘phenotypes’ [8, 9].

Despite a long history of clinical recognition, vocal cord dysfunction, inducible laryngeal obstruction, or paradoxical vocal fold motion [10] (hereafter VCD/ILO) remains an area in which “little is scientifically proven” [11]. There is agreement that VCD/ILO is a clinically impactful entity that can cause burdensome respiratory symptoms, particularly dyspnoea [12]. VCD/ILO may reach a prevalence of over 40% in some populations [4, 13–15], is associated with higher healthcare utilisation [16] and potentially harmful overtreatment including oral corticosteroid exposure.

The diagnosis is often delayed by years [17], leaving people with VCD/ILO to suffer the consequences of persistent symptoms, attacks of dyspnoea and side effects of ineffective therapies. Diagnosis can be delayed because clinicians do not recognise the patient’s presentation as VCD/ILO, thus, scaling clinical capability is a key aim, but heterogenous diagnostic approaches hamper this goal.

At a clinical level, the diagnostic process starts with a clinician suspecting VCD/ILO based on their knowledge of clinical features and associations. The next step is the performance of diagnostic tests. Progress in the recognition of VCD/ILO has been hindered by a lack of uniform diagnostic definitions and approaches. Considerable debate has surrounded the condition’s nomenclature [11, 18, 19]. However, diagnostic criteria and methodologies have received less attention. Different diagnostic methods yield heterogenous diagnostic rates [13] and there is a paucity of data to inform clinicians as to an optimum diagnostic approach. Halvorsen et al. [20] summarised key literature, emphasizing inducible aspects of VCD/ILO, but clinically important gaps remain, for example, specific criteria constituting laryngeal obstruction.

Diagnostic methodologies that are agreed upon are necessary to facilitate scientific progress and ultimately improve clinical management including scaling up clinical services. The Delphi method, predicated on group-consensus, is a methodology that may achieve agreement on important diagnostic issues. Prior approaches have not applied this consensus methodology to diagnosis of VCD/ILO.

We therefore detail a Delphi aiming to seek agreement by key stakeholders on a diagnostic approach to VCD/ILO.

## Study objective

The primary aim of this study is to establish consensus-based diagnostic criteria and methods for VCD/ILO.

Output from this process will be used to generate an expert consensus statement for publication. Information obtained may be used to create educational material to enhance awareness of this condition.

## Methods

The Delphi method is a common process of generating expert consensus [21–23], and purposively samples informants whose expertise and experience is likely to inform approaches to the problem in question [24]. It has the advantages of anonymity, avoiding group influences that can be present during in-person methods, and being more feasible than in-person formats with geographically diverse stakeholders [22]. There is no agreement on the optimum methodology for modified Delphi studies. Core features including number of rounds and feedback vary, and there is no EQUATOR network guideline for reporting.

It has the disadvantage that outlier opinions may be discounted, which may include opinions that may be innovative but may not be representative of mainstream opinion [24]. We will address this limitation by transparently listing all responses in appendices (except those which would uniquely identify participants), and publishing views that did not achieve consensus, if considered plausible by the steering committee.

A modified two-round Delphi will be performed. There is limited evidence that subsequent rounds achieve greater agreement and participant fatigue escalates after two rounds [22, 23], reducing participation. Findings will be validated by a workshop/focus-group (in-person and/or online participation). Results will be circulated, presented and discussed at dedicated sessions at two major subject-specific conferences in 2022 with the aims of exploring agreement, disagreement and obtaining consensus on this work. Both conferences (ILO conference in Bergen, Norway and VCD/ILO International Roundtable Meeting in Melbourne, Australia) will be attended by international experts in VCD/ILO and include attendance by almost all clinicians, academics and researchers active in the field.

### Ethics, dissemination and record retention

Institutional Review Board approval has been granted via the Monash Health Human Research Ethics Committee (HREC/80036/MonH-2021-285838, RES-21-0000665L). If substantial amendments are required, a variation will be submitted in writing to the HREC.

Records will be maintained in a secure password protected digital format conforming to the 2019 National Health and Medical Research Council Management of Data and Information in Research: A guide supporting the Australian Code for the Responsible Conduct of Research [25]. These will be held for 7 years and will be accessible only to authorised personnel.

The primary output will be a scientific publication. It is also envisaged that results will be presented at scientific conferences and may be used for educational purposes.

### Study schema

The Delphi will be performed via Qualtrics, a secure online survey platform hosted by Monash University. A survey invitation outlining the objectives of the study, its processes, and a participation link will be emailed to potential participants. A link to the patient information and consent form will be included in this email. By agreeing to start the survey participants will have been considered to have provided implied consent.

A candidate list of participants will be identified from a literature review. Authors who have published two or more original articles primarily concerning VCD/ILO diagnosis in the past 10 years will be included. Any authorship will qualify as scientific fields vary in their sequencing of author seniority. Authors presenting case studies, a single review article only, abstracts and articles not in English will not automatically be included in the initial list. A recent meta-analysis which included non-English publications found only one non-English article(2). It is unclear if authors who do not speak English could participate in this Delphi, because the study is being performed in English. The list will be presented to the steering committee, augmented by snowball sampling and personal contacts of the steering committee and will be agreed upon by a majority of the steering committee.

Participants will be offered group byline authorship of the resultant publication upon completion of both rounds of the Delphi. Participants may opt-out from group byline authorship, or the survey at any point. Participation to that point will be acknowledged in the publication.

Individuals with significant commercial conflicts of interest will be excluded.

A formal sample size calculation is not appropriate for a Delphi. The minimum sample size recommended in the literature is around 10 people [21, 26] but it is anticipated that the expert panel should be at least 25 individuals given heterogeneity of views in the literature, the number of authors in the literature, and the need to obtain a representative sample for validity given the variety of professions that diagnose and manage the condition [26].

Round 1 will examine clinical features and diagnostic approaches (S1 Appendix). Demographics of the Expert Panel will be collected: age, sex, number of articles published, years in practice, practice setting and specialty of practice in order to characterise panel composition, and the intent is to use this data descriptively. A review of diagnostic instruments has been undertaken by the first and last authors, extracting clinical features associated with VCD/ILO for validation by the expert panel. The search terms vocal cord dysfunction, inducible laryngeal obstruction, paradoxical vocal fold motion were used in PubMed and Google Scholar, and features were extracted from diagnostic instruments. These clinical features will be presented to participants for rating (on a Likert 1–5 scale, 1 least suggestive to 5 strongly suggestive), abstain and free-text comment(s).

Diagnostic methods will be explored by closed and open-ended questions, checklists as appropriate. Four clinical scenarios reflecting typical real-world patients have been developed by steering committee consensus and are included at the end of Round 1. Free text feedback will be used to ensure thematic saturation has been achieved in qualitative feedback in regards to how clinicians recognise and diagnose the condition.

The survey instrument will be tested with a small number of participants to check feasibility and understanding. Following dissemination, statements will be analysed and grouped thematically. Results of round 1 will be analysed by the study facilitator in consultation with the steering committee.

Round 2 will consist of statement agreement. In round 2, statements that did not generate consensus in round 1 will be presented to the expert panel for agreement using a five-point Likert scale (1 strongly disagree to 5 strongly agree). New statements from thematic analysis of Round 1 will be presented (anonymised). Feedback from Round 1 will be provided as percentage agreement (strongly agree, agree, neutral, disagree, strongly disagree) on statements.

During both rounds, each statement will have a free-text box to explore divergent responses and/or interpretation challenges. Some statements may be best elicited as categorical responses and these will be presented in an appropriate radio-button or tick-box format.

It is envisaged that the result of this Delphi will be presented in scientific conference(s) possibly as a workshop or in a roundtable format for validation.

Expert panel participant numbers will be recorded at each stage to produce a study flow chart. This will include–number of invited experts, number successfully contacted, number willing to participate, number returning surveys at each step, number excluded at each step.

During the survey/analysis phase of the Delphi, expert panel participants will remain anonymous. Anonymity will be achieved by expert panel participants being assigned a unique study ID number. For scientific presentation/publication, results will not contain any personal data which would enable an individual to be uniquely re-identified. During the workshop phase, participant anonymisation will not be possible. During presentation/publication, panel participant’s names will be listed. The draft publication will be circulated to Delphi survey participants for feedback before submission.

The study will be conducted by the study facilitator (Dr Paul Leong, Monash University, Melbourne, Australia). Roles include:

- Protocol development, ethics
- Managing participant invitations
- Developing and testing questionnaires
- Documenting study activity
- Implementing study rounds
- Study reporting

A steering committee will be established. It will consist of:

- Philip Bardin (Monash University, Melbourne, Australia)
- Mark Hew (Monash University, Melbourne, Australia)
- Anne Vertigan (John Hunter Hospital, Newcastle, Australia)
- Malcolm Baxter (Monash University, Melbourne, Australia)
- Debra Phyland (Monash University, Melbourne, Australia)
- Vanessa McDonald (University of Newcastle, Newcastle, Australia)
- Peter Gibson (University of Newcastle, Newcastle, Australia)
- James Hull (Royal Brompton Hospital, London, UK)
- Thomas Carroll (Brigham and Women’s Hospital, Boston, USA)
- An invited patient representative with lived experience of VCD/ILO.

The steering committee will:

- Provide an initial list of potential participants
- Facilitate communication between the study facilitator and experts if required
- Provide feedback on the round 1 questionnaire
- Guide facilitator in analysing the round 1 and round 2 proposals
- Participate in questionnaire testing
- Be invited to participate in the round 1 and 2 questionnaires
- Participate in study output finalisation

### Analysis

Statistical analysis will be performed in SPSS, R or GraphPad Prism. Normality will be judged with histograms and Shapiro-Wilk tests. Normally distributed variables will be summarised as mean +/- standard deviation. If non-Gaussian, data will be presented as median and interquartile range. Categorical data will be presented as number and percentage/proportions. Mean and/or median scores as appropriate will be generated for statements and used to order them and descriptive statistics will be produced from the demographic questionnaire. Qualitative free text responses will be coded and analysed thematically (e.g. with NVivo).

### Definition of consensus

Agreement will be defined as ≥ 70% [21, 27, 28] of the expert panel rating preferences as follows:

- When ≥70% of the expert panel indicates Likert scores of 4 or 5 (corresponding to “agree” or “strongly agree”, “important” or “very important” or similar) for a given statement, a positive consensus has been attained. For dichotomous questions, ≥70% of the expert panel indicating “yes” will be regarded as a positive consensus response.
- When ≥70% of the expert panel indicates either Likert scores of 1 or 2 (corresponding to “disagree” or “strongly disagree”, “not all important” or “low importance”, or similar) for a given statement, a negative consensus has been attained. For dichotomous questions, ≥70% of the expert panel indicating “no” will be regarded as a negative consensus response.
- When ≥70% of the expert panel indicates Likert 3 (“neutral”) for a given statement, a consensus for neutrality has been attained.
- When none of the three preceding definitions for consensus are met, the statement is classified as “controversial”.

Free text feedback fields will be inspected, and feedback will be analysed for possible item misinterpretation.

## Supporting information

S1 Appendix (PDF)

## References

[1] Dunglison R. The practice of medicine; or, a treatise on special pathology and therapeutics: In 2 volumes. Lea & Blanchard; 1842.

[2] Jackson C, Jackson C. Motor neuroses of the larynx. Dis Inj Larynx—Textb Stud Pract. 1942; 287–94.

[3] Christopher KL, Wood RP, Eckert RC, Blager FB, Raney RA, Souhrada JF. Vocal-Cord Dysfunction Presenting as Asthma. N Engl J Med. 1983;308: 1566–1570. doi: 10.1056/NEJM198306303082605 6406891

[4] Leong P, Ruane LE, Phyland D, Koh J, MacDonald MI, Baxter M, et al. Inspiratory vocal cord closure in chronic obstructive pulmonary disease. Eur Respir J. 2020; 1901466. 32029449 10.1183/13993003.01466-2019

[5] Daley CP, Ruane LE, Leong P, Lau KK, Low K, Hamza K, et al. Vocal Cord Dysfunction in Patients Hospitalised with Symptoms of Acute Asthma Exacerbation. Am J Respir Crit Care Med. 2019;200: 782–785. doi: 10.1164/rccm.201902-0396LE 31150268

[6] Patterson R, Schatz M, Horton M. Munchausen’s stridor: non-organic laryngeal obstruction. Clin Allergy. 1974;4: 307–310. doi: 10.1111/j.1365-2222.1974.tb01390.x 4426113

[7] Morris MJ, Christopher KL. Diagnostic Criteria for the Classification of Vocal Cord Dysfunction. Chest. 2010;138: 1213–1223. doi: 10.1378/chest.09-2944 21051397

[8] Leong P, Phyland DJ, Koo J, Baxter M, Bardin PG. Middle airway obstruction: phenotyping vocal cord dysfunction/inducible laryngeal obstruction(s) to progress diagnosis and management. Lancet Respir Med. 2022. doi: 10.1016/S2213-2600(21)00501-4 34973210

[9] Leong P, Al-Harrasi M, Carr B, Leahy E, Bardin PG, Barnes S. Vocal cord dysfunction/inducible laryngeal obstruction(s) mimicking anaphylaxis during SARS-CoV-2 (COVID-19) vaccination. J Allergy Clin Immunol Pract. 2022. doi: 10.1016/j.jaip.2022.02.025 35257958 PMC8896860

[10] Altman KW, Mirza N, Ruiz C, Sataloff RT. Paradoxical vocal fold motion: Presentation and treatment options. J Voice. 2000;14: 99–103. doi: 10.1016/s0892-1997(00)80099-5 10764121

[11] Christensen PM, Heimdal J-H, Christopher KL, Bucca C, Cantarella G, Friedrich G, et al. ERS/ELS/ACCP 2013 international consensus conference nomenclature on inducible laryngeal obstructions. Eur Respir Rev. 2015;24: 445–450. doi: 10.1183/16000617.00006513 26324806 PMC9487687

[12] Herth FJF, Schuhmann M. Vocal cord dysfunction or inducible laryngeal obstruction: whatever it is, it exists. Lancet Respir Med. 2017;5: 548–549. doi: 10.1016/S2213-2600(17)30222-9 28664857

[13] Lee J-H, An J, Won H-K, Kang Y, Kwon H-S, Kim T-B, et al. Prevalence and impact of comorbid laryngeal dysfunction in asthma: A systematic review and meta-analysis. J Allergy Clin Immunol. 2020;145: 1165–1173. doi: 10.1016/j.jaci.2019.12.906 31940470

[14] Low K, Lau KK, Holmes P, Crossett M, Vallance N, Phyland D, et al. Abnormal Vocal Cord Function in Difficult-to-Treat Asthma. Am J Respir Crit Care Med. 2011;184: 50–56. doi: 10.1164/rccm.201010-1604OC 21471099

[15] Lee JW, Tay TR, Paddle P, Richards AL, Pointon L, Voortman M, et al. Diagnosis of concomitant inducible laryngeal obstruction and asthma. Clin Exp Allergy. 2018;48: 1622–1630. doi: 10.1111/cea.13185 29870077

[16] Baxter M, Ruane L, Phyland D, Leahy E, Heke E, Lau KK, et al. Multidisciplinary team clinic for vocal cord dysfunction directs therapy and significantly reduces healthcare utilization. Respirology. 2019; resp.13520. doi: 10.1111/resp.13520 30884033

[17] Walsted ES, Famokunwa B, Andersen L, Rubak SL, Buchvald F, Pedersen L, et al. Characteristics and impact of exercise-induced laryngeal obstruction: an international perspective. ERJ Open Res. 2021;7: 00195–02021. doi: 10.1183/23120541.00195-2021 34195253 PMC8236618

[18] Bardin PG, Low K, Ruane L, Lau KK. Controversies and conundrums in vocal cord dysfunction. Lancet Respir Med. 2017;5: 546–548. doi: 10.1016/S2213-2600(17)30221-7 28664856

[19] Hull JH, Selby J, Sandhu G. “You Say Potato, I Say Potato”: Time for Consensus in Exercise-induced Laryngeal Obstruction? Otolaryngol Neck Surg. 2014;151: 891–892. doi: 10.1177/0194599814549529 25344097

[20] Halvorsen T, Walsted ES, Bucca C, Bush A, Cantarella G, Friedrich G, et al. Inducible laryngeal obstruction: an official joint European Respiratory Society and European Laryngological Society statement. Eur Respir J. 2017;50. doi: 10.1183/13993003.02221-2016 28889105

[21] Vernon W. The Delphi technique: A review. Int J Ther Rehabil. 2009;16: 69–76. doi: 10.12968/ijtr.2009.16.2.38892

[22] Hasson F, Keeney S, McKenna H. Research guidelines for the Delphi survey technique. J Adv Nurs. 2000;32: 1008–1015. 11095242

[23] Gossler T, Falagara Sigala I, Wakolbinger T, Buber R. Applying the Delphi method to determine best practices for outsourcing logistics in disaster relief. J Humanit Logist Supply Chain Manag. 2019;9: 438–474. doi: 10.1108/JHLSCM-06-2018-0044

[24] Edwards A, Webb H, Housley W, Beneito-Montagut R, Procter R, Jirotka M. Forecasting the governance of harmful social media communications: findings from the digital wildfire policy Delphi. Polic Soc. 2021;31: 1–19. doi: 10.1080/10439463.2020.1839073

[25] National Health and Medical Research. Management of Data and Information in Research: A guide supporting the Australian Code for the Responsible Conduct of Research. National Health and Medical Research Council; 2019. https://www.nhmrc.gov.au/sites/default/files/documents/attachments/Management-of-Data-and-Information-in-Research.pdf

[26] Trevelyan EG, Robinson PN. Delphi methodology in health research: how to do it? Eur J Integr Med. 2015;7: 423–428. doi: 10.1016/j.eujim.2015.07.002

[27] Suehs CM, Menzies-Gow A, Price D, Bleecker ER, Canonica GW, Gurnell M, et al. Expert Consensus on the Tapering of Oral Corticosteroids for the Treatment of Asthma. A Delphi Study. Am J Respir Crit Care Med. 2021;203: 871–881. doi: 10.1164/rccm.202007-2721OC 33112646

[28] Upham J, Lievre CL, Jackson D, Masoli M, Wechsler M, Price D. Late Breaking Abstract—Defining a severe asthma super-responder: findings from a Delphi process. Eur Respir J. 2020;56. doi: 10.1183/13993003.congress-2020.2610 34271216

