# Supplementary material for: Diagnosis of vocal cord dysfunction / inducible laryngeal obstruction—A Delphi study protocol
Source: PLoS One. 2022 Dec 29;17(12):e0279338. doi: 10.1371/journal.pone.0279338 (PMC9799289; doi:10.1371/journal.pone.0279338)
Supplement: S1 Appendix — (PDF) [file pone.0279338.s001.pdf]

APPENDIX 1 - VCD ILO Delphi Round 1

Start of Block: Welcome intro text

Intro Start Welcome to round 1 of the vocal cord dysfunction / inducible laryngeal obstruction(s) (VCD/ILO) Diagnosis Delphi.

The goal of this Delphi is to generate consensus on how experts diagnose VCD/ILO. The terms VCD and ILO will be used interchangeably.

This Delphi will take part in two rounds. This is the first round, and it is designed to understand clinical features you regard as important and diagnostic methods you use.

We compile responses and round 2 will consist of statements presented to you for rating in about one to two months.

Completing both rounds will be rewarded by your inclusion in the group authorship byline of the resulting publication. You can opt out if you wish.

Completing this survey implies acceptance of the Patient Information and Consent Form for this Delphi, viewable here. If you have concerns or questions, please contact the Principal Investigator, Dr Paul Leong, at paul.leong@monash.edu or +61 3 9594 2045. You may also contact the Human Research Ethics Coordinator at Monash Health at research@monashhealth.org or +61 3 9594 4611.

After demographic questions, there are three components. The first part presents clinical features of VCD/ILO. The second part asks about your approach to diagnostic tests. The third part presents scenarios.

This survey (round 1) is anticipated to take about 15-20 minutes.

End of Block: Welcome intro text

Start of Block: Demographics

*

Age What is your age (in years)?

____

Gender What is your gender?

- ○ Male (1)
- ○ Female (2)
- ○ X (Indeterminate/Intersex/Unspecified) (3)
- ○ Prefer not to say (4)

Diplomas Please indicate your degree(s). Please select all that apply.

- ☐ Medical degree (e.g. MBBS/MD or similar) (1)
- ☐ Nursing degree (e.g. BN or similar) (2)
- ☐ Speech pathology degree (e.g. BApplSci or similar) (3)
- ☐ Physiotherapy degree (e.g. BPhysio or similar) (4)
- ☐ Research higher doctorate (e.g. PhD/DPhil or similar) (5)
- ☐ Other (please specify) (6) ____

Page Break

Practice Environ Please indicate your practice environment. Please select all that apply.

- ☐ University/academic hospital (sometimes called tertiary or teaching hospital) (1)
- ☐ Regional hospital (2)
- ☐ Community (3)
- ☐ Private practice (4)
- ☐ Research (5)
- ☐ Other (please specify) (6) ____

Specialty Please indicate your practice specialty? Please select all that apply.

- ☐ Respiratory/Pulmonology (1)
- ☐ Allergy/Immunology (2)
- ☐ Ear/Nose/Throat Surgery (3)
- ☐ Paediatrics (4)
- ☐ Radiology (5)
- ☐ Speech language therapy/speech pathology (6)
- ☐ Physiotherapy (7)
- ☐ Nursing (8)
- ☐ Other (please specify) (9) ____

Patient ages Do you see adult, adolescent or paediatric patients? Please select all that apply.

- ☐ Adult (1)
- ☐ Adolescent (2)
- ☐ Pediatric (3)

*

Years Since Training How many years ago did you complete your clinical training?

____

Page Break

Years in VCD For how many years have you managed patients with VCD/ILO as a licensed/credentialed care provider (not including training)?

____

Pts per mo with VCD Approximately how many patients per month do you see with VCD/ILO?

____

VCD investigator Have you participated in, or been an investigator for studies of VCD/ILO?

- Yes (1)
- No (2)

Q15 How many scientific publications (journal articles) have you authored on VCD/ILO?

____

Q16 How many scientific publications (journal articles) have you authored in total (including VCD/ILO publications)?

____

VCD funding Have you ever received funding for VCD/ILO research?

- ◯ Yes (1)
- ◯ No (2)

VCD COI Do you have a financial conflict of interest with regards to VCD/ILO diagnosis?

- ◯ No (1)
- ◯ Yes (please specify) (2) ____

End of Block: Demographics

Start of Block: Section 1: Clinical Features

Intro clin feat
During this survey, VCD/ILO will be used interchangeably.

There are three components to this survey. The first part presents clinical features of VCD/ILO. The second part asks about your approach to diagnostic tests. The third part presents scenarios.

Clin feats
Section 1: Clinical features

A literature review has identified the following clinical features as being associated with VCD/ILO.

Please rate these features in terms of their importance in the diagnosis of VCD/ILO on the five-point scale below. You may also abstain. Please make any comments in the adjacent free text field.

	Importance	Free

							text
	Not at all important (1)	Low importance (2)	Neutral (3)	Important (4)	Very important (5)	Abstain (6)	Free text (1)

Symptoms are confined to the throat/upper chest. (1)	○	○	○	○	○	○	
It is difficult to breath past a certain point because of restriction. (2)	○	○	○	○	○	○	
Attacks are associated with throat tightness. (3)	○	○	○	○	○	○	
Breathlessness is worse on breathing in. (4)	○	○	○	○	○	○	
Attacks have specific triggers. (5)	○	○	○	○	○	○	
Exercise is a trigger. (6)	○	○	○	○	○	○	
Odours trigger attacks. (7)	○	○	○	○	○	○	

Breathing is worse with stress. (8)	○	○	○	○	○	○	
Attacks have rapid onset (seconds to minutes). (9)	○	○	○	○	○	○	
Attacks are associated with a change in voice. (10)	○	○	○	○	○	○	
On auscultation there is wheezing and/or stridor predominantly in the cervical region. (11)	○	○	○	○	○	○	

Page Break

Clin feats 2

Section 1: Clinical features

A literature review has identified the following clinical features as being associated with VCD/ILO.

Please rate these features in terms of their importance in the diagnosis of VCD/ILO on the five-point scale below. You may also abstain. Please make any comments in the adjacent free text field.

	Importance						Free text
	Not at all important (1)	Low importance (2)	Neutral (3)	Important (4)	Very important (5)	Abstain (6)	Free text (1)

Wheeze is absent during attacks. (1)	○	○	○	○	○	○	
Breathing is noisy during attacks. (2)	○	○	○	○	○	○	
If intubated during an attack, the pressures required for ventilation are remarkably low, relative to the severity of the attack. (3)	○	○	○	○	○	○	
Frustration that symptoms have been misunderstood. (4)	○	○	○	○	○	○	
Symptoms impact social life. (5)	○	○	○	○	○	○	
There has been no, or little therapeutic response to	○	○	○	○	○	○	

high inhaled corticosteroid or oral corticosteroids. (6)							
Pulmonary function testing shows flattening of the inspiratory loop of the flow-volume loops. (7)	○	○	○	○	○	○	
There are symptoms of laryngeal hypersensitivity (e.g. abnormal sensation in the throat including ache, itch, pain, or foreign body sensation and/or the inability to tolerate light pressure on the neck). (8)	○	○	○	○	○	○	
Cough. (9)	○	○	○	○	○	○	

Normal oxygen saturations during a clinically severe attack. (10)	○	○	○	○	○	○
Inhaled bronchodilators are ineffective or have relatively little effect. (11)	○	○	○	○	○	○

Page Break

VCD associations The following conditions have been proposed as being associated with VCD/ILO. Please rate how commonly they are associated with VCD/ILO on the five-point scale below. You may also abstain. Please make any comments in the adjacent free text field.

	Association frequency						Free text
	Never (1)	Rarely (2)	Sometimes / Neutral (3)	Often (4)	Always (5)	Abstain (6)	Free text (1)

Asthma (1)	○	○	○	○	○	○
Chronic obstructive pulmonary disease (2)	○	○	○	○	○	○
Reflux (3)	○	○	○	○	○	○
Chronic cough (4)	○	○	○	○	○	○
Smoking (5)	○	○	○	○	○	○
Irritable bowel syndrome (6)	○	○	○	○	○	○
Psychiatric diagnosis (7)	○	○	○	○	○	○

Chronic pain (8)	○	○	○	○	○	○

VCD assoc freetext Are there other clinical features or comorbidities of VCD/ILO that you would like to comment on?

____

End of Block: Section 1: Clinical Features

Start of Block: Section 2: Diagnostic tools

Intro diagtools
Section 2: Diagnostic tools

This section asks your opinion about diagnostic tools for VCD/ILO.

VCD DiagToolUse
Please indicate which of the following tools you use when diagnosing VCD/ILO. Please select all that apply. Please make any comments in the adjacent free text field.

Your responses to this question will enable or disable further questions on these tools in the later part of this survey.

	Tool use			Free text
	Yes - use (1)	No - do not use	Abstain (3)	Free text (1)

		(2)		
Laryngoscopy without provocation (1)	○	○	○	
Laryngoscopy with provocation (2)	○	○	○	
Spirometry with inspiratory and expiratory flow volume loops (3)	○	○	○	
Dynamic computed tomography of larynx (4)	○	○	○	
Questionnaires (5)	○	○	○	
Other (please use free text) (6)	○	○	○	

VCD ToolCanDx Please indicate which of the following tools, if abnormal, **can confirm** a diagnosis of VCD/ILO?

	Tool can diagnose if abnormal			Free text
	Yes - can confirm diagnosis (1)	No - cannot confirm diagnosis (2)	Abstain (3)	Free text (1)

Laryngoscopy without provocation (1)	○	○	○	
Laryngoscopy with provocation (2)	○	○	○	
Spirometry with inspiratory and expiratory flow volume loops (3)	○	○	○	
Dynamic computed tomography of larynx (4)	○	○	○	
Questionnaires (5)	○	○	○	
Other (please use free text) (6)	○	○	○	
None of the above (7)	○	○	○	

Page Break

VCD ToolCanMonitor Please indicate which of the following tools can be used for **disease monitoring** of VCD/ILO?

	Disease monitoring			Free text
	Yes - can monitor (1)	No - cannot monitor (2)	Abstain (3)	Free text (1)

Laryngoscopy without provocation (1)	○	○	○	
Laryngoscopy with provocation (2)	○	○	○	
Spirometry with inspiratory and expiratory flow volume loops (3)	○	○	○	
Dynamic computed tomography of larynx (4)	○	○	○	
Questionnaires (5)	○	○	○	
Other (please use free text) (6)	○	○	○	
None of the above (7)	○	○	○	

VCD Tool CanExclude Please indicate which of the following tools, **can rule out (i.e. exclude)** a diagnosis of VCD/ILO?

	Tool can diagnose if abnormal			Free text
	Yes - can exclude (1)	No - cannot exclude (2)	Abstain (3)	Free text (1)

Laryngoscopy without provocation (1)	○	○	○	
Laryngoscopy with provocation (2)	○	○	○	
Spirometry with inspiratory and expiratory flow volume loops (3)	○	○	○	
Dynamic computed tomography of larynx (4)	○	○	○	
Questionnaires (5)	○	○	○	
Other (please use free text) (6)	○	○	○	
None of the above (7)	○	○	○	

End of Block: Section 2: Diagnostic tools

Start of Block: Diagnostic Test Free Texts

IntroDiagTestQns The next component considers particular tests.

VCD Tool GoldStd Please indicate which one of the following tools is the **"gold standard"** for diagnosis of VCD/ILO?

- ○ Laryngoscopy without provocation (1)
- ○ Laryngoscopy with provocation (2)
- ○ Spirometry with inspiratory and expiratory flow volume loops (3)
- ○ Dynamic computed tomography of larynx (4)
- ○ Questionnaires (5)
- ○ Other (please use free text) (6) ____
- ○ None of the above (7)

VCD freetext spiro Please comment on the role of spirometry with inspiratory and expiratory flow loops in the diagnosis of VCD/ILO?

____

____

____

____

____

VCD freetext endosc Please comment on the role of laryngoscopy in the diagnosis of VCD/ILO?

____

Display This Question:

If Please indicate which of the following tools you use when diagnosing VCD/ILO. Please select all t... : Tool use = Laryngoscopy without provocation [Yes - use]

Or Please indicate which of the following tools you use when diagnosing VCD/ILO. Please select all t... : Tool use = Laryngoscopy with provocation [Yes - use]

VCD endosc_protocol You have indicated that you perform or use laryngoscopy. Can you please briefly describe how you do this?

Display This Question:

If Please indicate which of the following tools you use when diagnosing VCD/ILO. Please select all t... : Tool use = Laryngoscopy without provocation [Yes - use]

Or Please indicate which of the following tools you use when diagnosing VCD/ILO. Please select all t... : Tool use = Laryngoscopy with provocation [Yes - use]

VCD endosc_abnmovt What do you consider to be abnormal laryngeal movement? Please be as specific as possible: describe structures, phase of respiration and any other criteria such as percentage thresholds.

Display This Question:

If Please indicate which of the following tools you use when diagnosing VCD/ILO. Please select all t... : Tool use = Laryngoscopy without provocation [Yes - use]

Or Please indicate which of the following tools you use when diagnosing VCD/ILO. Please select all t... : Tool use = Laryngoscopy with provocation [Yes - use]

VCD endosc VC_insp Can you comment on the finding of vocal cord narrowing on INSPIRATION?

____

____

____

____

____

Display This Question:

If Please indicate which of the following tools you use when diagnosing VCD/ILO. Please select all t... : Tool use = Laryngoscopy without provocation [Yes - use]

Or Please indicate which of the following tools you use when diagnosing VCD/ILO. Please select all t... : Tool use = Laryngoscopy with provocation [Yes - use]

VCD endosc_VC_exp Can you comment on the finding of vocal cord (narrowing) on EXPIRATION?

____

____

____

____

____

Display This Question:

If Please indicate which of the following tools you use when diagnosing VCD/ILO. Please select all t... : Tool use = Laryngoscopy without provocation [Yes - use]

Or Please indicate which of the following tools you use when diagnosing VCD/ILO. Please select all t... : Tool use = Laryngoscopy with provocation [Yes - use]

VCD endoscp_SG_insp Can you comment on the finding of supraglottic narrowing on INSPIRATION?

____

____

____

____

____

Display This Question:

If Please indicate which of the following tools you use when diagnosing VCD/ILO. Please select all t... : Tool use = Laryngoscopy without provocation [Yes - use]

Or Please indicate which of the following tools you use when diagnosing VCD/ILO. Please select all t... : Tool use = Laryngoscopy with provocation [Yes - use]

VCD endosc_SG_exp Can you comment on the finding of supraglottic narrowing on EXPIRATION?

____

____

____

____

____

Display This Question:

If Please indicate which of the following tools you use when diagnosing VCD/ILO. Please select all t... : Tool use = Laryngoscopy with provocation [Yes - use]

VCD endosc_provo_sit
You have indicated you perform or use laryngoscopy with provocation. The next questions

concern provocation.

In which situations would you perform provocation?

____

____

____

____

____

Display This Question:

If Please indicate which of the following tools you use when diagnosing VCD/ILO. Please select all t... : Tool use = Laryngoscopy with provocation [Yes - use]

VCD endosc_provo_how Can you describe how you perform provocation? Which types of provoking agents do you use (for example, chemical(s) - which agent(s); exercise - how; hyperventilation - how)

____

____

____

____

____

Display This Question:

If Please indicate which of the following tools you use when diagnosing VCD/ILO. Please select all t... : Tool use = Laryngoscopy with provocation [Yes - use]

VCD emdpsc_normlaryx Can you describe what a positive (i.e. abnormal) laryngeal provocation test looks like?

____

____

Page Break

VCD ToolCT What is the role of **dynamic computed tomography of the larynx** in the diagnosis of VCD/ILO?

____

____

____

____

____

VCD ToolQuestionnair What is the role of **questionnaires** in the diagnosis of VCD/ILO?

____

____

____

____

____

VCD ToolFreeText Do you have any comments on VCD/ILO diagnostic tools?

____

____

____

____

____

End of Block: Diagnostic Test Free Texts

Start of Block: Section 3: Scenarios

Intro sect3
Section 3: Scenarios

This is the final section of this survey. It consists of clinical scenarios. Please consider and comment on the following scenarios.

Scenario1
Scenario 1

A 25-year-old person is referred with recurring dyspnoea, wheeze and dysphonia, sometimes provoked by strong smells and cold air. Spirometry including inspiratory and expiratory flow-volume loops is normal, mannitol broncho-provocation is negative (i.e. with no significant decrement in forced expiratory volume in 1 second). Laryngoscopy shows >50% sustained adduction (closure) of the cords on inspiration.

Scen1 InPractice Do you see patients like this in your practice?

○ Yes (1)

○ No (2)

Scen1 confidence How confident are you that this person has a diagnosis of VCD/ILO?

	Not confident at all (1)	Only slightly confident (2)	Somewhat confident (3)	Moderately confident (4)	Very confident (5)
Confidence (1)	○	○	○	○	○

Scen1 freetext Please provide any comments on this scenario.

____

____

____

____

Page Break

Scenario2
Scenario 2

An 18-year-old athlete is referred with episodic dyspnoea and dysphonia during competitive exercise. The person never smoked and asthma treatments have not helped. Spirometry including flow volume loops is normal at rest and after exercise. Mannitol broncho-provocation is negative (i.e. with no significant decrement in forced expiratory volume in 1 second). Laryngoscopy performed in the clinic, without exercise, is normal.

Scen2 inpractice Do you see patients like this in your practice?

○ Yes (1)

○ No (2)

Scen2 confidence How confident are you that this person has a diagnosis of VCD/ILO?

	Not confident at all (1)	Only slightly confident (2)	Somewhat confident (3)	Moderately confident (4)	Very confident (5)
Confidence (1)	○	○	○	○	○

Scen2 freetext Please provide any comments on this scenario.

____

____

____

____

____

Page Break

Scenario3
Scenario 3

A 30-year-old person is referred with episodic dyspnoea, wheeze and dysphonia. Symptoms do not respond to oral corticosteroid treatment. The patient has never smoked. They have severe asthma, with a forced expiratory volume in 1 second (FEV1) of 54% predicted with a significant (14%, 220mL) bronchodilator improvement. The patient is adherent to high dose inhaled corticosteroid/long acting beta agonist and receives parenteral monoclonal antibody treatment for asthma. Diffusing/transfer capacity of the lung for carbon monoxide is (i.e. DLCO or TLCO) is 85% predicted. Laryngoscopy shows >50% sustained adduction (closure) of the vocal cords on inspiration.

Scen3 Inpractice Do you see patients like this in your practice?

○ Yes (1)

○ No (2)

Scen3 confidence How confident are you that this person has a diagnosis of VCD/ILO?

	Not confident at all (1)	Only slightly confident (2)	Somewhat confident (3)	Moderately confident (4)	Very confident (5)
Confidence (1)	○	○	○	○	○

Scen3 freetext Please provide any comments on this scenario.

____

____

____

____

____

Page Break

Scenario4
Scenario 4

A 72-year-old person is referred with episodic dyspnoea, wheeze and dysphonia. They are a current tobacco smoker with a 50 pack year history. Significant emphysema is seen on chest computed tomography. Spirometry confirms severe chronic obstructive pulmonary disease, with a forced expiratory volume in 1 second (FEV1) of 43% predicted and FEV1/FVC ratio of 0.5. Diffusing/transfer capacity of the lung for carbon monoxide is (i.e. DLCO or TLCO) is 55% predicted. There is no significant change with bronchodilator administration. The patient is on maximal pharmacotherapy with a long acting beta agonist, long acting anti muscarinic and inhaled corticosteroid. Laryngoscopy shows sustained adduction (closure) of the vocal cords only on expiration.

Scen4 inpractice Do you see patients like this in your practice?

○ Yes (1)

○ No (2)

Scen4 confident How confident are you that this person has a diagnosis of VCD/ILO?

	Not confident at all (1)	Only slightly confident (2)	Somewhat confident (3)	Moderately confident (4)	Very confident (5)
Confidence (1)	○	○	○	○	○

Scen4 freetext Please provide any comments on this scenario.

____

____

____

____

____

Page Break

Scenario 5 **Scenario 5**

A 49 year old person with a history of generalised anxiety disorder reluctantly attended for coronavirus vaccination about one month prior to your review. Within 60 seconds of vaccine administration the patient reported dypsnoea, throat and chest tightness, noisy breathing, and experienced difficulty with phonation. The patient was immediately assessed for anaphylaxis but did not meet criteria for this diagnosis (no skin involvement, cardiovascular instability, or hypoxia). Salbutamol (albuterol) administration was mostly ineffective and the patient was discharged having largely recovered after a period of observation. The patient reports that symptoms have sometimes recurred. Laryngoscopy performed in the clinic, without provocation, is normal.

Scen5 inpractice Do you see patients like this in your practice?

○ Yes (1)

○ No (2)

Scen5 confidence How confident are you that this person has a diagnosis of VCD/ILO?

	Not confident at all (1)	Only slightly confident (2)	Somewhat confident (3)	Moderately confident (4)	Very confident (5)
Confidence (1)	○	○	○	○	○

Scen5 freetext Please provide any comments on this scenario.

____

____

____

____

____

Page Break

Scen6 VCDscenario
Scenario 6

Can you provide example(s) of other scenario(s) in which you would consider a person to have VCD/ILO?

____

____

____

____

____

Scen7 NotVCDscenario
Scenario 7

Can you provide example(s) of scenario(s) in which you would **never** consider a person to have VCD/ILO?

____

____

____

____

____

End of Block: Section 3: Scenarios
